# Screening of *Cucurbita maxima and Cucurbita moschata* Genotypes for Resistance Against *Meloidogyne arenaria, M. incognita, M. javanica*, and *M. luci*

**DOI:** 10.21307/jofnem-2019-057

**Published:** 2019-09-17

**Authors:** Gökhan Aydınlı, Ertan Sait Kurtar, Sevilhan Mennan

**Affiliations:** 1 Department of Crop and Animal Production, Bafra Vocational High School, Ondokuz Mayıs University, 55400 Samsun, Turkey; 2 Department of Plant Protection, Faculty of Agriculture, Ondokuz Mayıs University, 55139 Samsun, Turkey; 3 Department of Horticulture, Faculty of Agriculture, Selçuk University, 42130 Konya, Turkey

**Keywords:** *Cucurbita maxima*, *Cucurbita moschata*, management, *Meloidogyne* spp., pumpkin, reproduction, resistance, winter squash

## Abstract

The host response of fifteen winter squash (*Cucurbita maxima*) and five pumpkin (*Cucurbita moschata*) dihaploid genotypes to *Meloidogyne arenaria, M. incognita, M. javanica*, and *M. luci* was screened in pot experiments. Root galling and nematode reproduction were detected in all combinations of plant genotype and nematode species. Ten genotypes of *C. maxima* and three genotypes of *C. moschata* were considered highly resistant (<10% of the susceptible genotype) or moderately resistant (<50% of the susceptible genotype) to one or more *Meloidogyne* species based on nematode reproduction as a percentage of the most susceptible genotype. Genotypes 55CA15-A3 and G14-IP1 of *C. maxima* were highly resistant to *M. luci* and *M. arenaria*, respectively. Both 14BO01-O2 and G9-A4 genotypes of *C. moschata* were considered highly resistant to *M. arenaria*. However, these genotypes still allowed significant nematode reproduction because egg number per plant was higher than initial number of eggs used as inoculum, indicating that all genotypes were hosts.

Root-knot nematodes (RKN), *Meloidogyne* spp., are considered one of the major plant-parasitic nematodes worldwide (Jones et al., 2013). These obligate endoparasites infect roots and cause the formation of root galls which contain the modified feeding cells known as giant cells, which serve as the exclusive source of nutrients for nematode development (Caillaud et al., 2008). Due to nematode infection of roots, plant nutrient and water uptake are substantially reduced, resulting in above-ground symptoms such as stunting, yellowing, wilting, and yield losses (Caillaud et al., 2008; Moens et al., 2009). Additionally, RKN can increase the severity of plant damage when soilborne fungi such as *Fusarium* are present (Wang and Roberts, 2006).

*Meloidogyne arenaria, M. incognita*, and *M. javanica* have commonly been referred to as ‘major’ RKN species because they are globally distributed and are the most destructive nematode species infecting vegetable crops, particularly cucurbitaceous and solanaceous crops (Sikora and Fernández, 2005). In addition to these species, *M. luci* (formerly reported as *M. ethiopica*) is emerging as a significant problem for both open field and greenhouse vegetable crops in northern Turkey (Aydınlı and Mennan, 2016; Aydınlı, 2018). This species has been found on vegetables, fruit trees, and ornamental plants in America (Brazil, Chile, and Guatemala), Asia (Iran) and Europe (Greece, Italy, Portugal, Slovenia, and Turkey) (Conceição et al., 2012; Carneiro et al., 2014; Janssen et al., 2016; Gerič Stare et al., 2017; Aydınlı, 2018; Maleita et al., 2018). Therefore, *M. luci* is a potential threat for important vegetable crops such as cucumber, tomato, potato, and pepper because of its wide host range and wide geographical distribution (Strajnar et al., 2011; Carneiro et al., 2014; Gerič Stare et al., 2017; Aydınlı, 2018; Maleita et al., 2018).

Management of RKN in high-value crops was primarily based on chemical control throughout much of the past century (Nyczepir and Thomas, 2009). However, several nematicides, including methyl bromide, have been withdrawn or restricted during the last two decades due to their toxicity and adverse effects on the environment and human health (Ristaino and Thomas, 1997; Nyczepir and Thomas, 2009; Collange et al., 2011). In addition to the restrictions on nematicide use, increased interest in organically grown food has contributed to the development of alternative strategies for controlling nematode populations (Collange et al., 2011; Mashela et al., 2017). The use of resistant cultivars has been considered one of the most effective and inexpensive nematode control strategies. In intensive production systems such as greenhouse vegetable production, especially tomato, cucumber, and pepper in greenhouses of Turkey are frequently cultivated with limited crop rotation. RKN-resistance is commercially available for tomato and pepper, but not for cucumber (López-Gómez et al., 2016; Verdejo-Lucas and Talavera, 2019). Resistant rootstocks could be an effective method to solve this problem (Li and Chen, 2017). Grafting on *Cucurbita* spp. including *C. maxima, C. moschata, C. pepo*, and *C. ficifolia* is commonly used to increase yield and control soil-borne disease such as *Fusarium* wilt (Davis et al., 2008). For *C. maxima, C. moschata* and their interspecific hybrid (*C. maxima* x *C. moschata*), several studies were conducted to evaluate the levels of resistance against major RKN species (Sigüenza et al., 2005; Edelstein et al., 2010; Thies et al., 2010; Kokalis-Burelle and Rosskopf, 2011; Wilcken et al., 2013; Goreta Ban et al., 2014; López-Gómez et al., 2016; Giné et al., 2017; Verdejo-Lucas and Talavera, 2019). Although some genotypes may provide tolerance against RKN, resistance has not been identified (Verdejo-Lucas and Talavera, 2019). Most of experimental studies were performed on commercially available rootstocks, mainly *C. maxima* x *C. moschata* that is most widely used as a rootstock for cucumber, melon and watermelon (Sigüenza et al., 2005; Thies et al., 2010; Kokalis-Burelle and Rosskopf, 2011; Wilcken et al., 2013; Goreta Ban et al., 2014; López-Gómez et al., 2016; Giné et al., 2017; Verdejo-Lucas and Talavera, 2019). No study has been conducted to identify nematode resistance in the breeding germplasm lines of *C. maxima* and *C. moschata.*

The objective of this study was to determine the resistance reactions of fifteen winter squash (*C. maxima*) and five pumpkin (*C. moschata*) genotypes that could be used as rootstocks to manage the four RKN species (*M. arenaria, M. incognita, M. javanica*, and *M. luci*).

## Materials and methods

*Meloidogyne arenaria, M. incognita, M. javanica*, and *M. luci* used in the study were obtained from pot cultures in the Nematology Laboratory at the Ondokuz Mayıs University. The RKN isolates were originally established from single egg masses isolated from nematode-infested plants in vegetable greenhouses located in Samsun Province, Turkey and maintained on susceptible tomato (*Solanum lycopersicum*) cv. Falcon in pots (Aydınlı and Mennan, 2016). The species identity was confirmed by isoesterase phenotype. Nematode eggs used for inoculum were extracted from infested tomato roots using 0.52% sodium hypochlorite (NaOCl) (Hussey and Barker, 1973).

Fifteen winter squash (*C. maxima*) and five pumpkin (*C. moschata*) genotypes used in the study were developed by different dihaploidization techniques including irradiated pollen (Kurtar and Balkaya, 2010), anther culture (Kurtar et al., 2016) and ovule culture (Kurtar et al., 2018) (Table 1). Seeds were placed on wet filter paper in Petri dishes and germinated for 3 days at 24 ± 2°C in the dark. Pre-germinated seeds were sown singly in 300 ml pots filled with sterilized sandy soil. Pots were maintained in a plant growth room at 24 ± 2°C with a 16 hr/8 hr (light/dark) cycle. Two weeks after sowing, the seedlings were inoculated with 5000 eggs (Pi) at the second true leaf stages. Each RKN species-plant genotype combination was replicated four times and the experiment was repeated once. Pots were arranged in a completely randomized design in a plant growth room and plants were watered as needed during the experiments. Experiments were terminated 7 weeks after nematode inoculation. Plants were removed from pots and roots were carefully washed, weighed, and rated on a 0 (no galls) to 10 (100% galled) scale for gall index (GI) (Bridge and Page, 1980). Nematode eggs on each root were extracted with a 1% NaOCl solution by blender maceration and counted.

**Table 1.** Origin of winter squash (*Cucurbita maxima*) and pumpkin (*C. moschata*) genotypes used in this study.

Genotype	Origin	Source
*C. maxima*		
57SI21-IP1	57SI21	Kurtar and Balkaya (2010)
57SI21-IP2		Kurtar and Balkaya (2010)
57SI21-A3		Kurtar et al. (2016)
57SI21-A6		Kurtar et al. (2016)
57SI21-O7		Kurtar et al. (2018)
57SI21-O11		Kurtar et al. (2018)
57SI06-IP1	57SI06	Kurtar and Balkaya (2010)
57SI06-IP3		Kurtar and Balkaya (2010)
55CA06-A1	55CA06	Kurtar et al. (2016)
55CA06-A4		Kurtar et al. (2016)
55BA03-A1	55BA03	Kurtar et al. (2016)
55CA15-A2	55CA15	Kurtar et al. (2016)
55CA15-A3		Kurtar et al. (2016)
55CA15-O9		Kurtar et al. (2018)
G14-IP1	G14	Kurtar and Balkaya (2010)
*C. moschata*		
14BO01-O1	14BO01	Kurtar et al. (2018)
14BO01-O2		Kurtar et al. (2018)
G9-A4	G9	Kurtar et al. (2016)
G9-A5		Kurtar et al. (2016)
G9-O12		Kurtar et al. (2018)

All analyses were performed using the SAS statistical software (SAS Institute, Cary, NC). Prior to the analyses, data were log transformed [log10(*x* + 1)] to homogenize the variances, and then subjected to analysis of variance (ANOVA). The effect of plant genotype on root weight, GI, and eggs per gram root was determined, with each nematode species analyzed separately. The data of both the experiments were combined because there were no significant interactions between the experimental trials and plant genotype. The significance of differences between plant genotypes within each cucurbit species was separated using Tukey’s HSD tests (*P* < 0.05).

## Results

Although wide variation in reproduction of RKN species was observed, nematode egg production per plant (final density) was higher than initial egg number used as inoculum for all genotypes of both *Cucurbita* species. As a general trend, the genotypes 55BA03-A1 or 55CA15-A2 for *C. maxima* and the genotype G9-A5 for *C. moschata* exhibited the most susceptible host responses to all RKN species (Tables 2–5).

**Table 2.** Root weight, gall index (GI) and eggs per gram root of *Meloidogyne arenaria* on genotypes of *Cucurbita maxima* and *C. moschata* in pot experiments.

Genotype	Root weight (g)	GI^a^	Eggs/g root	Resistance designation^b^
*C. maxima*				
57SI21-IP1	3.90 ± 0.20 defg	7.88 ± 0.30 abc	16734 ± 1471 de	S
57SI21-IP2	2.80 ± 0.16 g	6.25 ± 0.16 e	18450 ± 2443 bcde	S
57SI21-A3	3.48 ± 0.38 efg	7.25 ± 0.16 cd	36042 ± 2827 a	S
57SI21-A6	3.46 ± 0.28 efg	8.75 ± 0.16 a	39911 ± 4924 a	S
57SI21-O7	4.44 ± 0.27 cdef	7.63 ± 0.32 bc	24815 ± 1376 abcd	S
57SI21-O11	4.80 ± 0.11 cd	6.50 ± 0.19 de	11768 ± 875 ef	MR
57SI06-IP1	4.63 ± 0.37 cde	7.75 ± 0.25 abc	29306 ± 4407 abc	S
57SI06-IP3	3.30 ± 0.32 fg	8.50 ± 0.19 ab	17156 ± 1408 cde	S
55CA06-A1	2.78 ± 0.18 g	6.13 ± 0.13 e	28110 ± 1632 abc	S
55CA06-A4	4.49 ± 0.25 cdef	7.50 ± 0.19 bc	16847 ± 1528 de	S
55BA03-A1	7.11 ± 0.15 a	7.63 ± 0.18 bc	33326 ± 3760 a	S
55CA15-A2	6.14 ± 0.43 ab	8.00 ± 0.00 abc	30655 ± 3574 ab	S
55CA15-A3	5.60 ± 0.27 bc	7.75 ± 0.16 abc	9430 ± 1265 f	MR
55CA15-O9	3.07 ± 0.21 g	6.13 ± 0.13 e	12384 ± 741 ef	MR
G14-IP1	3.09 ± 0.08 g	5.88 ± 0.23 e	2644 ± 190 g	HR
*C. moschata*				
14BO01-O1	2.99 ± 0.16 b	6.88 ± 0.30 b	27423 ± 2677 b	MR
14BO01-O2	5.91 ± 0.38 a	7.25 ± 0.16 b	6134 ± 790 c	HR
G9-A4	2.83 ± 0.22 b	4.75 ± 0.16 c	5474 ± 597 c	HR
G9-A5	2.68 ± 0.10 b	8.25 ± 0.16 a	62155 ± 5801 a	S
G9-O12	2.77 ± 0.19 b	7.00 ± 0.27 b	38177 ± 5127 b	S

Notes: Data are the mean ± standard error of two experiments (four replicates for each experiment). Statistical analyses of data were based on log10 (x + 1) transformed data. Means within a column followed by the same letters for each plant species are not significantly different according to Tukey's HSD test at *P*<0.05. _a_GI was based on a scale from 0 (no galls) to 10 (100% of roots galled) (Bridge and Page, 1980). _b_Resistance is based on reproduction relative to the most susceptible genotype (55BA03-A1 and 55CA15-A2 for *C*. *maxima* and G9-A5 for *C. moschata*). Reproduction <10% that of the susceptible genotype is considered highly resistant (HR), £50% is considered moderately resistant (MR), and >50% is considered susceptible (S).

**Table 3.** Root weight, gall index (GI) and eggs per gram root of *Meloidogyne incognita* on genotypes of *Cucurbita maxima* and *C. moschata* in pot experiments.

Genotype	Root weight (g)	GI^a^	Eggs/g root	Resistance designation^b^
*C. maxima*				
57SI21-IP1	5.68 ± 0.17 a	7.50 ± 0.27 bcde	12227 ± 746 fg	MR
57SI21-IP2	3.27 ± 0.26 cdef	7.50 ± 0.33 bcde	20687 ± 1219 cd	S
57SI21-A3	2.66 ± 0.15 ef	8.00 ± 0.27 abcd	23554 ± 1700 cd	S
57SI21-A6	2.91 ± 0.09 def	8.25 ± 0.25 abc	17028 ± 1154 de	S
57SI21-O7	5.71 ± 0.34 a	7.25 ± 0.25 cde	12047 ± 1478 fg	MR
57SI21-O11	4.25 ± 0.29 bc	7.13 ± 0.23 cde	9308 ± 514 g	MR
57SI06-IP1	3.40 ± 0.10 cdef	8.50 ± 0.19 ab	22673 ± 1636 cd	S
57SI06-IP3	3.61 ± 0.21 bcde	7.88 ± 0.13 abcd	39499 ± 1917 a	S
55CA06-A1	2.78 ± 0.17 ef	6.63 ± 0.18 e	16987 ± 1678 de	S
55CA06-A4	4.50 ± 0.29 ab	7.38 ± 0.26 bcde	25059 ± 446 bc	S
55BA03-A1	3.96 ± 0.28 bcd	7.88 ± 0.23 abcd	33905 ± 1255 ab	S
55CA15-A2	3.51 ± 0.22 bcde	8.00 ± 0.19 abcd	39663 ± 3164 a	S
55CA15-A3	3.30 ± 0.25 cdef	8.63 ± 0.18 a	14508 ± 928 fg	MR
55CA15-O9	2.68 ± 0.18 ef	7.00 ± 0.27 de	10282 ± 791 g	MR
G14-IP1	2.62 ± 0.17 f	8.50 ± 0.19 ab	18994 ± 1133 cd	S
*C. moschata*				
14BO01-O1	3.74 ± 0.54 b	8.25 ± 0.16 a	56002 ± 8086 ab	S
14BO01-O2	6.21 ± 0.20 a	8.00 ± 0.00 a	50951 ± 2998 ab	S
G9-A4	6.12 ± 0.38 a	8.13 ± 0.13 a	47032 ± 3124 b	S
G9-A5	5.82 ± 0.48 a	8.13 ± 0.13 a	45326 ± 3968 b	S
G9-O12	3.08 ± 0.20 b	8.13 ± 0.13 a	70915 ± 3829 a	S

Notes: Data are the mean ± standard error of two experiments (four replicates for each experiment). Statistical analyses of data were based on log10 (x + 1) transformed data. Means within a column followed by the same letters for each plant species are not significantly different according to Tukey's HSD test at *P*<0.05. _a_GI was based on a scale from 0 (no galls) to 10 (100% of roots galled) (Bridge and Page, 1980). _b_Resistance is based on reproduction relative to the most susceptible genotype (55BA03-A1 and 55CA15-A2 for *C*. *maxima* and G9-A5 for *C. moschata*). Reproduction <10% that of the susceptible genotype is considered highly resistant (HR), £50% is considered moderately resistant (MR), and >50% is considered susceptible (S).

**Table 4.** Root weight, gall index (GI) and eggs per gram root of *Meloidogyne javanica* on genotypes of *Cucurbita maxima* and *C. moschata* in pot experiments.

Genotype	Root weight (g)	GI^a^	Eggs/g root	Resistance designation^b^
*C. maxima*				
57SI21-IP1	5.55 ± 0.32 ab	6.13 ± 0.13 cd	6244 ± 426 ef	MR
57SI21-IP2	3.66 ± 0.23 cdef	5.50 ± 0.57 de	21206 ± 2011 ab	S
57SI21-A3	3.55 ± 0.48 def	7.25 ± 0.16 abc	13853 ± 1405 c	S
57SI21-A6	2.73 ± 0.12 f	7.50 ± 0.27 ab	8830 ± 678 de	MR
57SI21-O7	3.74 ± 0.28 cdef	3.63 ± 0.18 f	4359 ± 589 fg	MR
57SI21-O11	4.35 ± 0.18 bcde	4.88 ± 0.13 e	3119 ± 347 g	MR
57SI06-IP1	3.11 ± 0.16 ef	8.50 ± 0.19 a	5621 ± 357 f	MR
57SI06-IP3	5.31 ± 0.41 ab	7.38 ± 0.18 ab	14219 ± 562 bc	S
55CA06-A1	4.73 ± 0.37 abcd	6.88 ± 0.13 bc	16197 ± 1360 abc	S
55CA06-A4	5.96 ± 0.23 a	7.00 ± 0.00 bc	18338 ± 1490 abc	S
55BA03-A1	5.11 ± 0.36 abc	7.50 ± 0.19 ab	18348 ± 753 abc	S
55CA15-A2	4.17 ± 0.23 bcde	7.25 ± 0.16 abc	21806 ± 933 a	S
55CA15-A3	3.58 ± 0.20 def	7.50 ± 0.19 ab	17962 ± 1074 abc	S
55CA15-O9	3.28 ± 0.26 ef	6.63 ± 0.18 bc	13072 ± 1244 c	S
G14-IP1	2.87 ± 0.17 f	7.13 ± 0.13 abc	5729 ± 298 f	MR
*C. moschata*				
14BO01-O1	4.03 ± 0.42 b	7.25 ± 0.16 b	28924 ± 3039 d	MR
14BO01-O2	7.06 ± 0.22 a	8.00 ± 0.00 a	53259 ± 2769 b	S
G9-A4	7.30 ± 0.16 a	8.00 ± 0.00 a	51721 ± 5434 b	S
G9-A5	6.31 ± 0.32 a	7.75 ± 0.16 ab	71450 ± 4601 a	S
G9-O12	3.09 ± 0.09 b	6.25 ± 0.16 c	40865 ± 3240 bc	S

Notes: Data are the mean ± standard error of two experiments (four replicates for each experiment). Statistical analyses of data were based on log10 (x + 1) transformed data. Means within a column followed by the same letters for each plant species are not significantly different according to Tukey's HSD test at *P*<0.05. _a_GI was based on a scale from 0 (no galls) to 10 (100% of roots galled) (Bridge and Page, 1980). _b_Resistance is based on reproduction relative to the most susceptible genotype (55BA03-A1 and 55CA15-A2 for *C*. *maxima* and G9-A5 for *C. moschata*). Reproduction <10% that of the susceptible genotype is considered highly resistant (HR), £50% is considered moderately resistant (MR), and >50% is considered susceptible (S).

**Table 5.** Root weight, gall index (GI) and eggs per gram root of *Meloidogyne luci* on genotypes of *Cucurbita maxima* and *C. moschata* in pot experiments.

Genotype	Root weight (g)	GI^a^	Eggs/g root	Resistance designation^b^
*C. maxima*				
57SI21-IP1	7.48 ± 0.34 a	7.75 ± 0.31 abcd	19071 ± 2846 d	MR
57SI21-IP2	5.38 ± 0.36 bcde	7.88 ± 0.13 abcd	21923 ± 809 cd	MR
57SI21-A3	6.45 ± 0.42 abc	8.50 ± 0.19 ab	35623 ± 3365 bc	S
57SI21-A6	6.33 ± 0.49 abc	8.75 ± 0.16 a	29438 ± 5060 bcd	S
57SI21-O7	5.73 ± 0.34 abcd	8.13 ± 0.13 abc	36444 ± 4583 bc	S
57SI21-O11	4.28 ± 0.29 cde	5.63 ± 0.18 e	4856 ± 416 e	MR
57SI06-IP1	6.26 ± 0.53 abcd	7.75 ± 0.16 abcd	26005 ± 3796 cd	S
57SI06-IP3	7.18 ± 0.71 ab	7.63 ± 0.26 bcd	31562 ± 4911 bcd	S
55CA06-A1	3.51 ± 0.28 ef	7.13 ± 0.13 d	20797 ± 1271 cd	MR
55CA06-A4	5.88 ± 0.46 abcd	8.00 ± 0.19 abcd	32246 ± 5771 bcd	S
55BA03-A1	7.30 ± 0.28 ab	8.38 ± 0.18 ab	53064 ± 3789 a	S
55CA15-A2	7.59 ± 0.43 a	8.50 ± 0.19 ab	47280 ± 2876 ab	S
55CA15-A3	4.96 ± 0.25 cde	5.50 ± 0.19 e	2671 ± 310 f	HR
55CA15-O9	2.90 ± 0.19 f	5.75 ± 0.16 e	6089 ± 312 e	MR
G14-IP1	5.09 ± 0.37 cde	7.25 ± 0.25 cd	7871 ± 1099 e	MR
*C. moschata*				
14BO01-O1	5.53 ± 0.48 ab	7.63 ± 0.18 a	16800 ± 2570 b	MR
14BO01-O2	7.31 ± 0.50 a	7.50 ± 0.19 a	15548 ± 2290 b	MR
G9-A4	6.02 ± 0.53 ab	7.50 ± 0.19 a	22700 ± 1262 ab	S
G9-A5	4.45 ± 0.52 bc	7.75 ± 0.16 a	34525 ± 3971 a	S
G9-O12	3.42 ± 0.14 c	6.50 ± 0.19 b	27980 ± 1912 ab	S

Notes: Data are the mean ± standard error of two experiments (four replicates for each experiment). Statistical analyses of data were based on log10 (x + 1) transformed data. Means within a column followed by the same letters for each plant species are not significantly different according to Tukey's HSD test at *P*<0.05. _a_GI was based on a scale from 0 (no galls) to 10 (100% of roots galled) (Bridge and Page, 1980). _b_Resistance is based on reproduction relative to the most susceptible genotype (55BA03-A1 and 55CA15-A2 for *C*. *maxima* and G9-A5 for *C. moschata*). Reproduction <10% that of the susceptible genotype is considered highly resistant (HR), £50% is considered moderately resistant (MR), and >50% is considered susceptible (S).

In response to *M. arenaria* inoculation, the genotype G14-IP1 had the lowest number of eggs per gram root (2644 eggs) and was significantly different (*P* < 0.05) from other genotypes of *C. maxima* (Table 2). Additionally, nematode reproduction on the genotypes 57SI21-IP1, 57SI21-O11, 57SI06-IP3, 55CA06-A4, 55CA15-A3, and 55CA15-O9 was lower than on 55BA03-A1 and 55CA15-A2, the most susceptible genotypes for *C. maxima* (*P* < 0.05). Reproduction of *M. arenaria* on G14-IP1 was 7.9% that of the susceptible genotype, 55BA03-A1; whereas, reproduction on genotypes 55CA15-A3, 57SI21-O11, and 55CA15-O9 was 28.3%, 35.3%, and 37.2% that of 55BA03-A1, respectively, indicating moderate resistance. Gall indices on the *C. maxima* genotypes inoculated with *M. arenaria* ranged from 5.88 (G14-IP1) to 8.75 (57SI21-A6). In contrast to nematode reproduction, galling rates of the genotypes 57SI21-IP1, 57SI06-IP3, 55CA06-A4, and 55CA15-A3 were similar to most susceptible genotypes (*P* > 0.05). Of the *C. moschata* genotypes, the lowest nematode reproduction was observed on the genotypes 14BO01-O2 and G9-A4, with <10% the reproduction observed on the most susceptible genotype G9-A5 (Table 2). However, 14BO01-O2 showed a significantly higher gall index compared to genotype G9-A4.

*Meloidogyne incognita* eggs per gram root were lower on 57SI21-IP1, 57SI21-O7, 57SI21-O11, 55CA15-A3, and 55CA15-O9 than on the other genotypes of *C. maxima* (Table 3). These five genotypes exhibited a moderate resistance response to *M. incognita* relative to reproduction on the most susceptible genotypes 55BA03-A1 and 55CA15-A2, but none of the *C. maxima* genotypes showed a high level of resistance to *M. incognita*. Only the genotype 55CA06-A1 showed a significantly lower gall index compared to the most susceptible genotypes. In contrast to other *Meloidogyne* species tested on the genotype G9-A5 of *C. moschata*, *M. incognita* produced numerically fewer eggs than the other genotypes (Table 3). Only two genotypes of *C. moschata* (G9-A4 and G9-A5) showed a low level resistance to *M. incognita,* with reproduction ranging from 64 to 66% that of the most susceptible genotype G9-O12 (Table 3). Severe root galling was observed on all the genotypes and there were no differences among the genotypes (*P* > 0.05).

The reproduction of *M. javanica* was significantly lower on 57SI21-IP1, 57SI21-A6, 57SI21-O7, 57SI21-O11, 57SI06-IP1, and G14-IP1 compared to that of other genotypes of *C. maxima* (Table 4). These six genotypes supported <50% the reproduction observed on the most susceptible genotypes 55BA03-A1 and 55CA15-A2, indicating moderate resistance levels. On the genotypes of *C. maxima* inoculated with *M. javanica*, GI values ranged from 3.68 (57SI21-O7) to 8.50 (57SI06-IP1). Of the *C. moschata* genotypes, only 14BO01-O1 produced significantly fewer eggs than the other genotypes and showed a moderate resistance level to *M. javanica* with reproduction being 40.5% that of the most susceptible genotype G9-A5. However, GI values on both genotypes were similar.

The reproduction of *M. luci* on the eight most resistant genotypes of *C. maxima* ranged from 2671 (55CA15-A3) to 26005 (57SI06-IP1) eggs per gram root and was significantly lower than the most susceptible genotypes 55BA03-A1 and 55CA15-A2 (Table 5). Of these eight genotypes, 55CA15-A3 showed the highest level of resistance with egg production being 5% that of the most susceptible genotype 55BA03-A1, whereas other genotypes, except 57SI06-IP1, supported <50% the nematode reproduction of the most susceptible genotypes 55BA03-A1 and 55CA15-A2. The GI values of *C. maxima* genotypes inoculated with *M. luci* ranged from 5.50 to 8.75 (Table 5). Root galling on the genotypes 57SI21-O11, 55CA15-A3, and 55CA15-O9 was lower than on the remaining genotypes (*P* < 0.05). Of *C. moschata* genotypes, 14BO01-O1 and 14BO01-O2 supported significantly lower number of eggs per gram root compared to the most susceptible genotype G9-A5 (Table 5). Both genotypes supported <50% nematode reproduction to that of the genotype G9-A5, indicating moderate resistance levels. The genotype G9-O12 showed a significantly lower gall index compared to other genotypes of *C. moschata*.

## Discussion

Twenty *Cucurbita* genotypes having potential use as rootstocks or for breeding programs were evaluated to identify resistance levels against different RKN species in pot experiments. The screening of these genotypes for resistance is of particular interest in overcoming the lack of available genetic sources to control RKN populations. Resistance is defined as the ability of a plant to significantly reduce nematode reproduction and is a relative concept because the level of nematode resistance in a plant genotype is compared to the nematode reproduction on a susceptible host (Hussey and Janssen, 2002). In RKN resistance, a highly resistant genotype supports little or no nematode reproduction (<10% of the susceptible genotype), whereas a moderately resistant plant allows intermediate levels of reproduction (<50% of the susceptible genotype) (Hadisoeganda and Sasser, 1982; Hussey and Janssen, 2002; Cortada et al., 2008).

Although nematode reproduction is often used as the primary indicator of nematode resistance, resistance to RKN can also be evaluated based on root galling (Thies and Levi, 2007; Edelstein et al., 2010; Mukhtar et al., 2013; Liu et al., 2015). Gall formations on roots indicate the successful induction of nematode feeding sites that are essential for nematode development. However, evaluation of host resistance based on root galling may be misleading because gall formation is not always followed by successful nematode development and reproduction (López-Gómez et al., 2015). Galling of roots indicates nematode damage caused by RKN; however, it is not necessarily a quantitative measure for host-plant resistance when not strongly correlated with nematode reproduction. There was not a clear correspondence between galling and egg production in this study. For example, the *C. maxima* genotypes 57SI06-IP1 and G14-IP1 inoculated with *M. javanica* showed the highest gall indices but produced low numbers of egg per gram root. In contrast to these genotypes, 57SI21-IP2 had a low gall index and high nematode reproduction. Moreover, in some plant genotypes, significant differences were observed in reproduction even though the plants had similar galling when inoculated with the same nematode species. For example, galling rates on 55CA06-A1 and 55CA15-O9 were similar for *M. arenaria*, whereas nematode reproduction rates were two-fold higher on 55CA06-A1 than on 55CA15-O9.

Root galling and nematode reproduction were observed on all plant genotype and nematode species combinations, indicating that none of the genotypes were immune to nematode infection. However, ten genotypes of *C. maxima* and three genotypes of *C. moschata* were considered highly resistant or moderately resistant to one or more *Meloidogyne* species based on nematode reproduction as a percentage of the most susceptible genotype. Of these genotypes, a highly resistant reaction (<10% of the susceptible genotype) was found on 55CA15-A3 and G14-IP1 of *C. maxima* and 14BO01-O2 and G9-A4 of *C. moschata.* The genotype 55CA15-A3 was highly resistant to *M. luci* but moderately resistant to *M. arenaria* and *M. incognita*. However, this genotype was highly susceptible to *M. javanica*. Genotypes G14-IP1, 14BO01-O2, and G9-A4 were highly resistant to *M. arenaria* and, except G9-A4, were also moderately resistant to *M. luci*. Moreover, the genotype G14-IP1 responded as moderately resistant to *M. javanica*. Only 57SI21-O11 showed moderate resistance to all *Meloidogyne* species tested. Genotypes 57SI21-IP1, 55CA15-O9, and 14BO01-O1 were each susceptible to either *M. arenaria, M. javanica*, and *M. incognita*, respectively, but moderately resistant to the remaining the three species. The genotype 57SI21-O7 responded as moderately resistant to *M. incognita* and *M. javanica*, and as susceptible to *M. arenaria* and *M. luci*. Genotypes 57SI21-IP2, 57SI21-A6, 57SI06-IP1, and 55CA06-A1 classified as moderately resistant were restricted to one species, and these genotypes were susceptible to the other three species. Of these genotypes, 57SI21-IP2 and 55CA06-A1 inoculated with *M. luci*, and 57SI21-A6 and 57SI06-IP1 inoculated with *M. javanica* were considered moderately resistant.

Many studies have been conducted to evaluate the potential of the commercial squash hybrid rootstocks (*C. moschata* x *C. maxima*) for the management of RKN but the genotypes displayed susceptible reactions, having significantly high nematode reproduction (Sigüenza et al., 2005; Thies et al., 2010; Kokalis-Burelle and Rosskopf, 2011; Goreta Ban et al., 2014; López-Gómez et al., 2016; Giné et al., 2017; Verdejo-Lucas and Talavera, 2019). Among the genotypes of *C. maxima* and *C. moschata* tested in this study, highly and moderately resistant genotypes were identified when compared to the most susceptible genotypes. However, even the most resistant genotypes (55CA15-A3, G14-IP1, 14BO01-O2, and G9-A4) allowed significant egg production, which was 1.6-7.2× the initial number of eggs used as inoculum. Nevertheless, cultivation of these genotypes would benefit succeeding crops because they will reduce nematode population build-up in comparison with other susceptible hosts (López-Gómez et al., 2015, 2016; Talavera-Rubia et al., 2018). These resistant genotypes could be combined with other management tools such as biofumigation, solarization, and biological agents to further suppress nematode populations. The important point to remember is that relative resistance level in a genotype can change depending on the susceptible genotype (Cortada et al., 2008). Therefore, further research is needed to determine if the genotypes found to be resistant in this study are significantly more resistant than commercial melon and pumpkin cultivars.
